# Endemicity of *Opisthorchis viverrini* Liver Flukes, Vietnam, 2011–2012

**DOI:** 10.3201/eid2001.130168

**Published:** 2014-01

**Authors:** Vo The Dung, Jitra Waikagul, Bui Ngoc Thanh, Dung Thi Vo, Duy Nhat Nguyen, K. Darwin Murrell

**Affiliations:** Research Institute for Aquaculture No. 3, NhaTrang, Vietnam (V.T. Dung, D.T. Vo, D.N. Nguyen);; Mahidol University, Bangkok, Thailand (J. Waikagul);; Research Institute for Aquaculture No. 1, Bac Ninh, Vietnam (B.N. Thanh);; University of Copenhagen, Copenhagen, Denmark (K.D. Murrell)

**Keywords:** zoonoses, *Opisthorchis viverrini*, parasites, liver, liver fluke, fish, metacercariae, Vietnam

**To the Editor:** Fishborne zoonotic trematodes are highly prevalent in many Asian communities (*1*,*2*). Although presence of the liver fluke *Clonorchis sinensis* is well documented in Vietnam (*3*), evidence of the presence of the more common liver fluke of Southeast Asia, *Opisthorchis viverrini,* is only circumstantial. Surveys of human fecal samples have frequently reported *O. viverrini* fluke eggs in humans in southern and central Vietnam (*4*); however, identifications based on fecal eggs are notoriously unreliable for differentiating species of liver and intestinal flukes (*5*). The few reports of surgical recovery of adult *O. viverrini* flukes from humans do not eliminate the possibility of infection having been acquired during travel in neighboring fluke-endemic countries. 

Metaceraria from fish in the Mekong Delta have been tentatively identified as *Opisthorchis* spp., but this identity has not been confirmed (*6*). Specific identification is necessary for an understanding of the liver fluke diversity in Vietnam, especially because *O. lobatus* flukes, a related species that infects ducks, have been reported from nearby Laos (*7*).

To clarify the status of fishborne liver flukes in Vietnam, during 2011–2012, we conducted a survey for liver fluke metacercariae in fish from Phu Yen Province. We selected this province because the local populations have a strong preference for raw fish and because previous surveys of human fecal samples conducted there indicated high prevalence of fishborne parasites (*4*). We chose to investigate metacercariae in fish to avoid the uncertainty of identifications based on fecal eggs and because of the availability of recent molecular methods for species identification of *Opisthorchis* fluke metacercariae (*7*). 

Fish were collected from Tuy Hoa City and from the districts of Hoa Xuan Dong, Tuy An, and Song Hinh; these 3 districts are areas of large aquaculture production of freshwater fish. Fresh fish from ponds, rice fields, rivers, and swamps were purchased at local markets from April 2011 through March 2012. The fish sellers provided information about the source of the fish (e.g., type of water body). Fish were transported live with mechanical aeration to the Research Institute for Aquaculture No. 3 in Nha Trang, where they were examined for metacercariae by use of whole individual fish pepsin digestion (*8*). 

Recovered metacercariae were examined microscopically, and those identified morphologically as *Opisthorchis* spp. flukes (*9*) were isolated. A subset of these metacercariae were fixed in 70% alcohol and examined by PCR and sequence analysis of the CO1 gene (*7*) at the Department of Helminthology, Mahidol University, Bangkok. For the purpose of obtaining adult worms, 3 hamsters were inoculated with the *Opisthorchis* metacercariae (15, 30, or 45 metacercariae/hamster). The adult worms were recovered from the infected hamsters 25–30 days after infection and were fixed and stained for morphologic determination of species (*10*).

A total of 4 fish species were infected with *O. viverrini* metacercariae (Technical Appendix Table 1). Metacercariae prevalence was highest (28.1%) among crucian carp (*Carasius auratus*). Specific identification was confirmed by morphologic appearance of adult worms recovered from hamsters (Figure) and PCR and sequence analysis of the partial metacercarial CO1 gene, amplified by CO1-OV-Hap-F&R primers (*7*). Infected fish originated predominantly from so-called wild water (i.e., swamps, rice fields, rivers). The prevalence of *O. viverrini* metacercariae in crucian carp varied seasonally (Technical Appendix Table 2).

**Figure:**
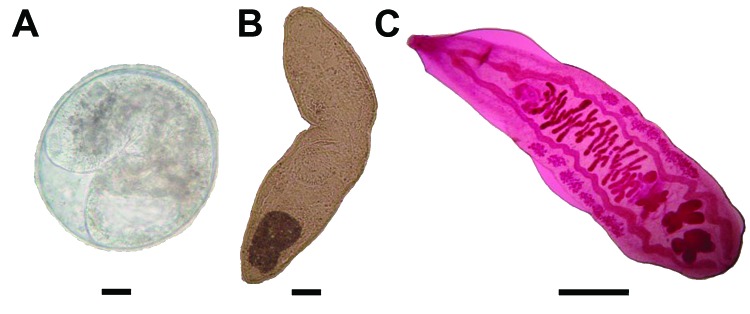
Morphologic appearance of different stages of *Opisthorchis viverrini* flukes. A) Encysted metacercariae. Scale bar indicates 30 μ. B) Metacercariae released from cyst. Scale bar indicates 30 μ. C) Carmine-stained adult worm from experimentally infected hamster. Scale bar indicates 1 mm.

Crucian carp are cultured in some countries but not in Vietnam. However, the high prevalence and mean intensity of *O. viverrini* metacercariae (28.3 metacercariae/fish) is of public health concern because wild species such as crucian carp are often eaten raw, marinated, or lightly cooked. In contrast, infected barb (*Puntius brevis*) and rasbora (*Rasbora* spp.) fish (Technical Appendix Table 1) are not eaten raw. However, barb fish are invasive in farm fish ponds and can persist as a self-recruiting species; the presence of barb is an indication that pond management is insufficient to prevent invasive species of fish. Furthermore, barb fish are often fed to farm cats, which are major reservoir hosts for fishborne liver and intestinal trematodes. Infections (prevalence 8.3%) in snakehead fish (*Channa* spp.) also represent a food safety risk, because snakehead fish are cultured in Vietnam and are sometimes eaten raw or inadequately cooked. In addition to *O. viverrini* flukes, metacercariae of the zoonotic intestinal flukes *Centrocestus formosanus, Haplorchis taichui*, *and H. yokogawi* were recovered from snakehead and barb fish (Technical Appendix Table 1), all of which are common throughout Southeast Asia (*1*).

The results of this study demonstrate that the human liver fluke *O. viverrini* is endemic to Vietnam and that it is being naturally transmitted to fish species that are often consumed raw or inadequately cooked. For determination of the prevalence, distribution, and epidemiology of *O. viverrini* flukes in fish, humans, and reservoir hosts (e.g., cats and dogs), these results need to be extended, especially because aquaculture is a growing industry in Vietnam.

Technical Appendix Prevalence and intensity of *Opisthorchis viverrini* fluke metacercariae in fish collected in several districts of Phu Yen Province, Vietnam, and in crucian carp collected monthly in An My commune, Tuy An district, Vietnam.
