# Supplementary material for: Endemicity of Opisthorchis viverrini Liver Flukes, Vietnam, 2011–2012
Source: Emerg Infect Dis. 2014 Jan;20(1):152–4. doi: 10.3201/eid2001.130168 (PMC3884709; doi:10.3201/eid2001.130168)
Supplement: Technical Appendix — Prevalence and intensity of Opisthorchis viverrini fluke metacercariae in fish collected in several districts of Phu Yen Province, Vietnam, and in crucian carp collected monthly in An My commune, Tuy An district, Vietnam. [file 13-0168-Techapp-s1.pdf]

Article DOI: http://dx.doi.org/10.3201/eid2001.130168

# Endemicity of *Opisthorchis viverrini* Liver Flukes, Vietnam, 2011–2012

## Technical Appendix

Technical Appendix Table 1. Prevalence and intensity of *Opisthorchis viverrini* fluke metacercariae in fish collected in several districts of Phu Yen province,Vietnam

| Fish species | Collection site district/ commune | Prevalence, % (No. infected/total no. examined) | Infection intensity (metacercariae/fish) | Type of water body | Other zoonotic metacercaria infections* |
|---|---|---|---|---|---|
| Crucian carp *(Carasius auratus)* | Tuy An /An My | 28.1 (72/256) | 28.2 ±29.5 | Swamp | None seen |
| Snakehead (*Channa* spp.) | Dong Hoa / Hoa Xuan Dong | 8.3 (1/12) | 1.0** | Rice field | *Centrocestus. formosanus* |
| Rasbora (*Rasbora* spp.) | Dong Hoa / Hoa Xuan Dong | 4.3 (1/23) | 2.0 | Rice field | None seen |
| Barb (*Puntius brevis*) | Dong Hoa / Hoa Xuan Dong | 50.0 (1/2) | 1.0±1.4 | Pond | *Haplorchis. taichui* and *H. yokogawai* |
| | Tuy An/Chi Thanh | 60.0 (3/5) | 2.7 ± 0.6 | River | *C. formosanus. H. taichui* and *H. yokogawai* |
| | Tuy An/An My | 100.0† (1/1) | 2.0 | Swamp | *H. taichui* and *H. yokogawai* |

*No determination of prevalence or intensity was made.
†Based on 1 infected fish.

Technical Appendix Table 2. Prevalence of *Opisthorchis viverrini* fluke metacercariae in crucian carp collected monthly in An My commune, Tuy An district, Vietnam

| Date | No. fish examined/no. fish infected | % Fish infected |
|---|---|---|
| 2011 | | |
| Apr | 83/0 | 0 |
| May | 17/5 | 29.4 |
| Jun | 21/3 | 14.3 |
| Nov | 41/6 | 14.6 |
| 2012 | | |
| Jan | 38/2 | 5.3 |
| Mar | 56/56 | 100 |
